# The Effect of Vaccination Coverage and Climate on Japanese Encephalitis in Sarawak, Malaysia

**DOI:** 10.1371/journal.pntd.0002334

**Published:** 2013-08-08

**Authors:** Daniel E. Impoinvil, Mong How Ooi, Peter J. Diggle, Cyril Caminade, Mary Jane Cardosa, Andrew P. Morse, Matthew Baylis, Tom Solomon

**Affiliations:** 1 Liverpool University Climate and Infectious Disease of Animals Group, Department of Epidemiology and Population Health, Institute of Infection and Global Health, University of Liverpool, Neston, Cheshire, United Kingdom; 2 Brain Infections Group, Department of Clinical Infection, Microbiology and Immunology, Institute of Infection and Global Health, University of Liverpool, Merseyside, Liverpool, United Kingdom; 3 Department of Paediatrics, Sibu Hospital, Sibu, Sarawak, Malaysia; 4 Lancaster Medical School, Faculty of Health and Medicine, Lancaster University, Lancaster, United Kingdom; 5 Department of Geography and Planning, School of Environmental Sciences, University of Liverpool, Merseyside, Liverpool, United Kingdom; 6 Institute of Health and Community Medicine, Universiti Malaysia Sarawak, Kota Samarahan, Sarawak, Malaysia; 7 The National Consortium for Zoonosis Research, Neston, Cheshire, United Kingdom; 8 Walton Centre NHS Foundation Trust, Fazakerley, Liverpool, Merseyside, United Kingdom; The George Washington University Medical Center, United States of America

## Abstract

**Background:**

Japanese encephalitis (JE) is the leading cause of viral encephalitis across Asia with approximately 70,000 cases a year and 10,000 to 15,000 deaths. Because JE incidence varies widely over time, partly due to inter-annual climate variability effects on mosquito vector abundance, it becomes more complex to assess the effects of a vaccination programme since more or less climatically favourable years could also contribute to a change in incidence post-vaccination. Therefore, the objective of this study was to quantify vaccination effect on confirmed Japanese encephalitis (JE) cases in Sarawak, Malaysia after controlling for climate variability to better understand temporal dynamics of JE virus transmission and control.

**Methodology/principal findings:**

Monthly data on serologically confirmed JE cases were acquired from Sibu Hospital in Sarawak from 1997 to 2006. JE vaccine coverage (non-vaccine years vs. vaccine years) and meteorological predictor variables, including temperature, rainfall and the Southern Oscillation index (SOI) were tested for their association with JE cases using Poisson time series analysis and controlling for seasonality and long-term trend. Over the 10-years surveillance period, 133 confirmed JE cases were identified. There was an estimated 61% reduction in JE risk after the introduction of vaccination, when no account is taken of the effects of climate. This reduction is only approximately 45% when the effects of inter-annual variability in climate are controlled for in the model. The Poisson model indicated that rainfall (lag 1-month), minimum temperature (lag 6-months) and SOI (lag 6-months) were positively associated with JE cases.

**Conclusions/significance:**

This study provides the first improved estimate of JE reduction through vaccination by taking account of climate inter-annual variability. Our analysis confirms that vaccination has substantially reduced JE risk in Sarawak but this benefit may be overestimated if climate effects are ignored.

## Author Summary

Japanese encephalitis (JE) virus is a mosquito-borne virus which is the leading cause of viral encephalitis in Asia. The cumulative attack rate for human JE cases is seemingly low (2-cases/10^5^/year); however, epidemics are focal and intense, being concentrated in children 1 to 15 years of age and highly clustered spatially. Despite the availability of a vaccine against JE virus (JEV), a significant number of JE cases still occur in Asia. Increases in disease cases may occur in some countries, such as Indonesia and Bangladesh, due to increasing rice cultivation, pig husbandry and lack of vaccination or vector control programs in these areas. However, given the sensitivity of JEV transmission to climate through impacts on the mosquito vectors, changes in climate may increase or decrease JEV transmission. Clearly, JE vaccination has substantially reduced JE cases, but it is not clear if climate has any confounding impact on our ability to estimate vaccine effect. We found that approximately one quarter of the reduction in JE cases since vaccination was introduced might be attributable to climatically unfavourable years for the disease, rather than the effect of vaccination. Vaccination studies involving climate-sensitive diseases may be made more accurate by taking climate effects into account.

## Introduction

Japanese encephalitis (JE) is a mosquito-borne disease which is the leading cause of viral encephalitis across Asia with approximately 70,000 cases a year and 10,000 to 15,000 deaths [1], [2]. There are two transmission patterns of JEV in three climatic zones [3], [4]: in temperate climate zones such as such as Japan, Korea and mainland China and Taiwan, and sub-tropical climate zones such as Nepal, and northerly areas of India, Thailand and Vietnam, JEV exhibits an epidemic or outbreak transmission pattern, characterized by a well-defined seasonal peak. In tropical climate zones such as Malaysia, Indonesia, southerly areas of Vietnam and Thailand, JEV exhibits an endemic transmission characterized by sporadic human cases throughout the year with seasonal peaks. Generally, it is considered that the prevailing transmission cycles and the observed seasonality are a function of irrigation practices, ambient temperature and bird migration patterns in different regions [5].

Longitudinal investigations into the influence of temperature and rainfall on the occurrence of serologically confirmed human JE cases have been conducted in temperate environments with epidemic JEV transmission, with studies generally showing that high temperatures lead to high numbers of JE cases, while rainfall associations are more variable [6]–[9]. To date no known parallel studies have been undertaken in tropical countries with JEV-endemic transmission. In addition, the influence El Niño/La Niña-Southern Oscillation (ENSO), a major modifier of climate in the tropics [10], on JEV transmission has received relatively little attention [5] despite its impact on other members of the Flavivirdae family, such as dengue [11]–[15]. Given that Malaysia, Indonesia and most of the Philippines usually are the first to experience ENSO-related impacts [16], investigation in these areas is warranted.

JE incidence varies widely over time, partly because of inter-annual climate variability effects on mosquito vector abundance. This makes it more complex to assess the effects of a vaccination programme because more or less climatically-favourable years could also contribute to a change in incidence post-vaccination. For example, a study in Taiwan looking at the association between climate, vaccination rate and JE occurrence, only during the post-vaccination period, found that the abundance of cases was still under the influence of climate [8]. Furthermore, given the impact of vaccination on confirmed JE cases, any association between JE cases and meteorological variables may be masked by the vaccination programme. Therefore, this study looks at Sarawak Malaysia, a tropical environment with endemic JEV transmission, during the pre- and post-vaccination period, with the objective of determining the impact of JE vaccination after controlling for meteorological variables and assessing the relationship between meteorological variables and confirmed JE cases. We test the hypotheses that: 1) temperature, rainfall and ENSO are associated with confirmed JE cases in Sibu, Malaysia after controlling for vaccination coverage year and 2) the estimated benefit of mass vaccination is adjusted when account is taken of climate conditions.

## Material and Methods

### Study area

All JE cases in this study originated from central Sarawak an area with approximately 650,000 people (180,000 children ≤12 years). Sarawak has a generally stable equatorial climate that is hot and humid throughout the year, with two monsoon seasons: a northeast monsoon season with heavy rainfall occurring from November to March and a southeast monsoon season with relatively less rainfall occurring from May/June to September [17]. All cases of encephalitis in the region are referred to Sibu Hospital. With the introduction of the formalin-inactivated mouse derived JE vaccine (Biken, Japan), a significant reduction in cases has been observed [18], [19].

### Vaccine administration

The JE vaccine is provided for free under the Malaysian National Immunisation Programme and the recommended JE immunization interval schedule in Sarawak is: the primary series at 9 and 10 months with subsequent booster at 18 months, 4.5, 8, 11 and 15 years; one injection of 0.5 mL is given at each interval [19]–[21]. However, in practice we are reasonably certain that the majority of children in our study received at least two doses of the vaccine between 9 and 12 months.

### Ethics statement

The original data collection of the study was approved by the Director of Health for Sarawak and the Ethics Committee of the Liverpool School of Tropical Medicine (Liverpool, UK). All data analyzed were anonymized by removing any potential unique identifiers before conducting the analysis. Informed consent was obtained from each child's parent or guardian before samples were taken [18].

### Data collection

The data on JE cases were obtained through a prospective study in Sibu Hospital and a JE surveillance programme set up by the Sarawak Health Department since 1997 [18], [19]. Briefly, patients with suspected JE were enrolled into the hospital-based surveillance study. Paired sera and paired CSF for each patient were considered to be the ideal specimen set. However, in reality the complete specimen set was not obtained from many cases, from whom we usually obtained a single serum and a CSF specimen. Specimens were tested for JEV specific immunoglobulin-type M by MAC-ELISA (Venture Technologies Sdn Bhd, Malaysia), which distinguishes IgM elicited by JEV from that elicited by dengue viruses [22], [23]. The sensitivity, specificity, positive predictive value and negative predictive value of the test used were 83%, 99%, 0.98 and 0.92, respectively for CSF and 91%, 95%, 0.92 and 0.94, respectively for serum [23]. All cases of encephalitis with specific IgM to JEV in serum and/or CSF were considered to have been infected recently with JEV and classified as confirmed JE cases. It is important to note that by the time a JE infection leads to encephalitis there is usually no virus in the periphery and thus a “confirmatory test” looking for evidence of virus or virus genome is not useful.

Monthly mean, maximum, minimum and range temperature (°C) and mean rainfall (cm/month) for central Sarawak was acquired from the KNMI Climate Explorer [24] based on the Climate Research Unit [25] dataset. The weather coverage window we used was latitude: 3.731–1.205° and longitude: 111.078–115.236° (see Supplemental Material, Figure S1). We used the updated CRU TS 3.1 dataset (1901–2009) at 0.5° (∼55.5 km) spatial resolution [26]. The ENSO index used here is the Southern Oscillation Index (SOI), air pressure difference calculated between Tahiti and Darwin, Australia [27]. Monthly SOI was acquired from the NOAA Climate Prediction Center (CPC) [28] via KNMI Climate Explorer.

### Statistical analysis

We fitted a Poisson log-linear regression model to the time series of monthly incident counts using the R open source software environment (www.r-project.org). We conducted the analysis in 3 phases:

Since it is well established that for JEV transmission there is a lag effect of meteorological variables prior to initiation of pathogen transmission [6]–[9], the initial phase in our analysis was to correlate JE cases with climate variables and identify temporal lags. We explored our data using Pearson's correlation coefficient. Since JEV transmission is thought to occur in Malaysia year-round in epizootic cycles [5], we assessed lag times up to 12 months.

In the second phase of our study, the monthly number of serologically confirmed JE cases was modelled using a Poisson regression model. There was no over-dispersion in our analysis; therefore, we found no need to set the scale parameter to a Pearson χ^2^ statistic. In our model, we adjusted for seasonality, trend effects associated with JE cases and lag effects of the meteorological variables, and used time series diagnostics (autocorrelation of standardised residuals) to check the assumption of uncorrelated residual variation.

We examined the relationships between the JE cases, vaccine, meteorological variables and a linear time trend. Fourier terms up to the second harmonic were included in the model to account for seasonality in JE cases. An assumed full model that adjusts for the effects of all the explanatory variables is:
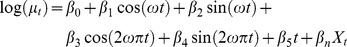
where 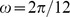 the annual periodicity in JE cases, *t* is an index of time given as a sequence of numbers (i.e. 1,2,3…‥n), *X_t_* is some covariate process (i.e. vaccine, temperature, rainfall and SOI) with associated temporal lags.

To select and retain variables that significantly contributed to the improvement of the model fit; we used a stepwise approach using Akaike's Information Criterion (AIC) as a measure of relative goodness of fit, where smaller values represents better fits. Risk ratios (RRs) derived from the estimated regression parameters from the final model illustrate the associations between the meteorological determinants and confirmed JE cases. We attempted to identify statistical interactions with all combination of seasonality, trend, vaccine coverage and meteorological variables, but none of the combinations were significant. We also determined the adjusted McFadden's pseudo R^2^ given by:
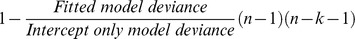
where 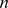 is the sample size and 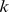 is the number of parameters.

We used the monthly data from the 10-year surveillance period in Sarawak from April 1997 to December 2006 where suspected JE cases were serologically confirmed as JE positive or negative. This dataset was used to test the association between JE cases, vaccine and meteorological variables in Sarawak and develop our model. We lagged vaccine coverage by two months to account for the interval between the first and second vaccination. This made clinical and immunological sense since the protection the vaccine provides does not reach clinically acceptable protective efficacy until the first two doses (given 1-month apart) are provided (Centers for Disease Control and Prevention 1993). Though we had three temperature variables (mean, maximum and minimum), we only used minimum temperature in the final model since it had the strongest fit and including mean and maximum temperature would be redundant. Each lagged variable of SOI was individually and iteratively assessed with temperature, rainfall and vaccine and the final model was selected based on the models with lower AIC value. We omitted the trend (i.e. timeindex) from the model because the downward trend in JE cases over the 10-year study period is likely attributed to the vaccination program. Also, vaccine coverage year and trend (i.e. timeindex) were found to be moderately correlated with each other (Pearson correlation coefficient = 0.859, p<0.001); therefore, vaccine coverage year was retained in the model, while trend was removed and not considered further.

We developed two models: a vaccine only model (vaccine and seasonality) and an observed climate and vaccine model (vaccine, seasonality, minimum temperature, rainfall and SOI).

In the third and final phase, we used the observed climate and vaccine model developed in the second analysis phase to predict the number of JE cases that would have been seen if there was no vaccine coverage throughout the 10-year surveillance and if there was vaccine coverage throughout the 10-year surveillance period.

### Vaccine effectiveness sensitivity analysis

We also conducted sensitivity analysis using different vaccine effectiveness values from 0 to 100% to see the range in risk reduction for different models. We used models with the variables: i) vaccine only, and; ii) vaccine, minimum temperature, rainfall and SOI. A study of children in Thailand vaccinated with two doses of the formalin-inactivated mouse derived JEV vaccine (Biken, Japan) showed that the vaccine had a 91% vaccine efficacy [29], [30] ; therefore, we assumed that the vaccine strategy being used in Malaysia had at least a 91% effectiveness for the target population, Sarawak children ≤12 years of age.

## Results

### JE cases

**Figure 1:**
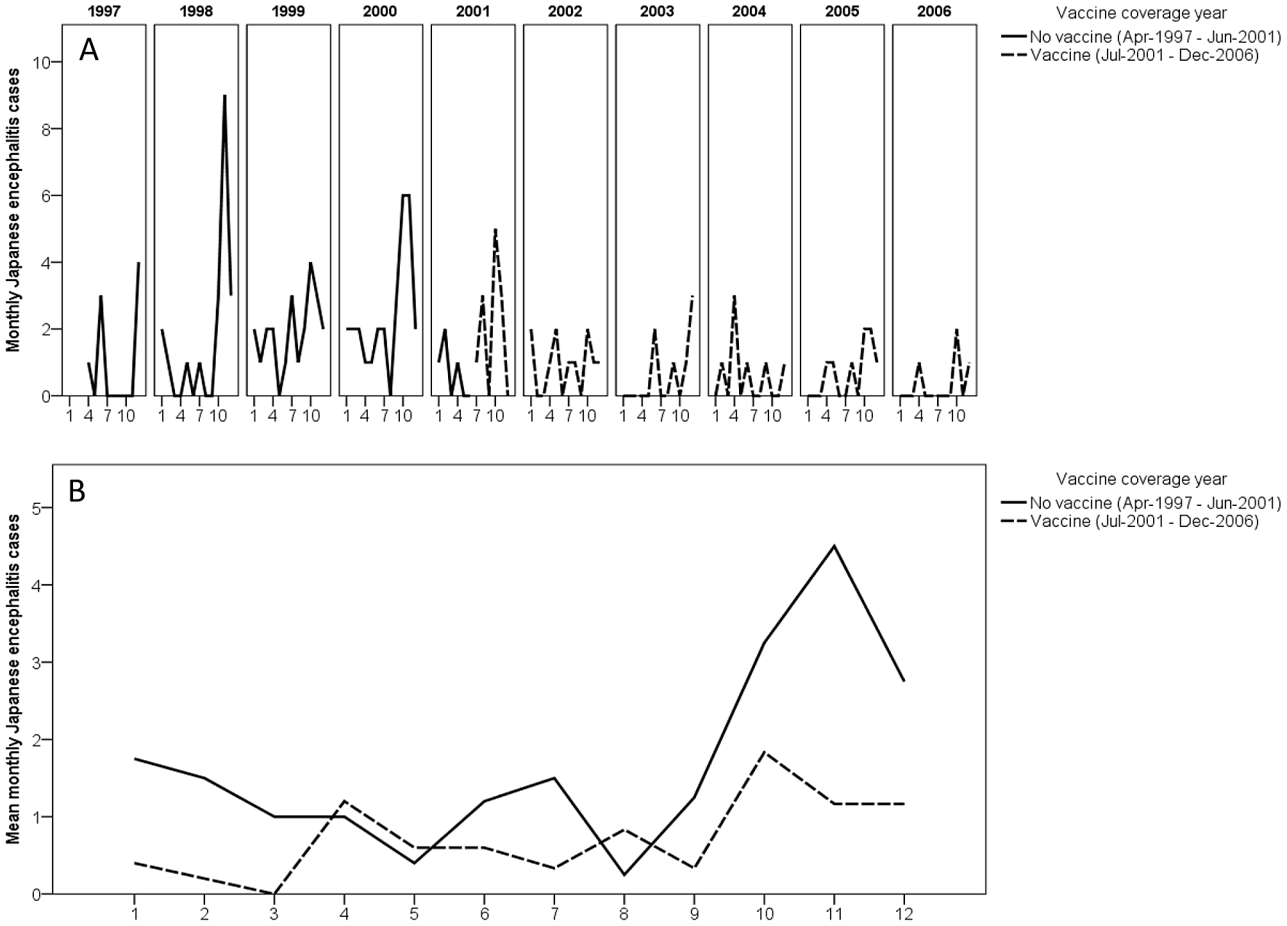
Time series (A) and seasonality (B) of confirmed Japanese Encephalitis cases from Sarawak, Malaysia from April 1997 to December 2006.

One-hundred-and-thirty-three serologically confirmed JE cases were identified from April 1997 to December 2006 (10-year period), 84 before and 49 after the vaccine period of July 2001. There were relatively high levels of JEV transmission for four seasons prior to the incorporation of JE vaccine into the expanded programme of immunization (EPI) in the state of Sarawak in July 2001 (Figure 1A). After the vaccination program, JE cases dropped substantially (Figure 1A). The majority of JE cases generally occurred in the 4^th^ quarter of the year (October to December) (Figure 1B).

### Correlation to JE cases

There was temporal variation in climate variables and JE cases (see Supplemental Material, Figure S2). The mean, maximum and minimum temperatures were correlated with JE cases at 5 to 7-months lag; minimum temperature at 6-month lag showed the strongest correlation with JE cases; and rainfall was only correlated with JE cases at 1-month lag (see Supplemental Material, Table S1).

We selected vaccine at 2-months lag, minimum temperature at 6-months lag, rainfall a 1-month lag to be used in further analysis since they provided a better fit than other variables and lags (data not shown). Due to the persistence of the ENSO, where its impact on climate [31], and subsequently disease risk [32], can be felt for a whole year in the tropics, we iteratively assessed up to 12 lags of SOI variables. SOI at 6-months lag were used in the final model.

### Poisson model fitting

Table 1 shows the association between vaccine at 2-months lag and JE cases after controlling for seasonality (vaccine only model); Table 2 shows the association between vaccine coverage at 2-months lag, minimum temperature at 6-months lag, rainfall at 1-month lag, and SOI at lag 6-month lag, and JE cases after controlling for seasonality (observed climate and vaccine model). All variables were significantly associated with JE cases in the different models. The observed climate and vaccine model had a higher adjusted pseudo-R^2^ and lower Akaike information criterion (AIC = 300.196, adjusted pseudo-R^2^ = 0.377) than the vaccine only model (Akaike information criterion = 317.398, adjusted pseudo-R^2^ = 0.278) suggesting the former is a “better” model. There was no significant autocorrelation in JE cases. Trend was not significant when included in the model with vaccine.

**Table 1:** Multivariable estimate of JE risk ratio for vaccine coverage year, controlling for seasonal periodicity using Poisson regression.

Variables	RR/β (95% CI of RR)	Std. Error	p-value^a^
**Vaccine year (Jul 01–Dec 06) at 2-month lag**	0.391^b^ (0.273, 0.560)	0.1838	<0.001
**Non-vaccine year (Apr 97–Jun 01) at 2-month lag**	1^c^	.	.
**cos12**	−0.288^d^	0.1420	0.043
**cos6**	−0.190^d^	0.1313	0.148
**sin12**	−0.500^d^	0.1297	<0.001
**sin6**	0.391^d^	0.1313	0.003
**Intercept**	0.398^d^	0.1142	<0.001

RR = risk ratio;

a Based on Wald chi-square test;

b Risk Ratio;

c Reference.

d β-value.

Akaike information criterion = 317.398, Adjusted Pseudo-R^2^ = 0.278. cos12 and sin12 models annual periodicity; cos6 and sin6 models biannual periodicity. Pseudo- R^2^ is based on 1 minus the deviance ratio between the full model vs. the Intercept only model adjusting for the number of explanatory terms in a model (1 – (Full model _DEV_/Intercept only model _DEV_) * ((n-1)/(n-k-1))), where n is the sample size and k is the number of explanatory terms.

**Table 2:** Multivariable estimate of the JE risk ratio for vaccine coverage year, different weather variables and Southern Oscillation Index (SOI), controlling for seasonal periodicity using Poisson regression.

Variables	RR/β (95% CI of RR)	Std. Error	p-value^a^
**Vaccine year (Jul 01–Dec 06) at 2-month lag**	0.548^b^ (0.368, 0.818)	0.2039	0.003
**Non-vaccine year (Apr 97–Jun 01) at 2-month lag**	1^c^	.	.
**Minimum temperature (°C) at 6-months lag**	2.016^b^ (1.269, 3.204)	0.2363	0.003
**Rainfall (cm/month) at 1-month lag**	1.027^b^ (1.007, 1.047)	0.0099	0.007
**SOI at 6-months lag**	1.404^b^ (1.147, 1.718)	0.1031	0.001
**cos12**	−0.311^d^	0.1558	0.046
**cos6**	−0.183^d^	0.1324	0.167
**sin12**	−0.213^d^	0.1596	0.181
**sin6**	0.239^d^	0.1402	0.088
**Intercept**	−15.422^d^	5.0538	0.002

RR = risk ratio;

a Based on Wald chi-square test;

b Risk Ratio;

c Reference.

d β-value.

Akaike information criterion = 300.196, Adjusted Pseudo-R^2^ = 0.377. cos12 and sin12 models annual periodicity; cos6 and sin6 models biannual periodicity. Pseudo- R^2^ is based on 1 minus the deviance ratio between the full model vs. the Intercept only model adjusting for the number of explanatory terms in a model (1 – (Full model _DEV_/Intercept only model _DEV_) * ((n-1)/(n-k-1))), where n is the sample size and k is the number of explanatory terms.

No interaction effects amongst the meteorological variables or vaccine variable were observed in our model suggesting no differential response of JE cases to the aforementioned variables.

For the vaccine only model, there seems to be an approximately 61% decrease in JE cases after the implementation vaccination programme (post July 2001) relative to prior the implementation (pre July 2001). In contrast, for the observed climate and vaccine models, the estimated decrease is approximately 45%.

The observed climate and vaccine models also suggest that a 1-unit increase in minimum temperature at a 6-month lag, rainfall at 1-month lag, and SOI at 6-month lag may be associated with approximately 100% (2-fold), 3% and 40% increases in risk of JE in Sarawak respectively. To better understand the effect of the scale of variability in temperature, rainfall and SOI on JE cases we also calculated the relative risk using the difference between the lower and upper quartiles of the explanatory variable. There was on average a 41%, 53% and 66% increase in JE risk for the upper-lower quartile difference for temperature, rainfall and SOI, respectively (see Supplemental Material, Table S2).

**Figure 2:**
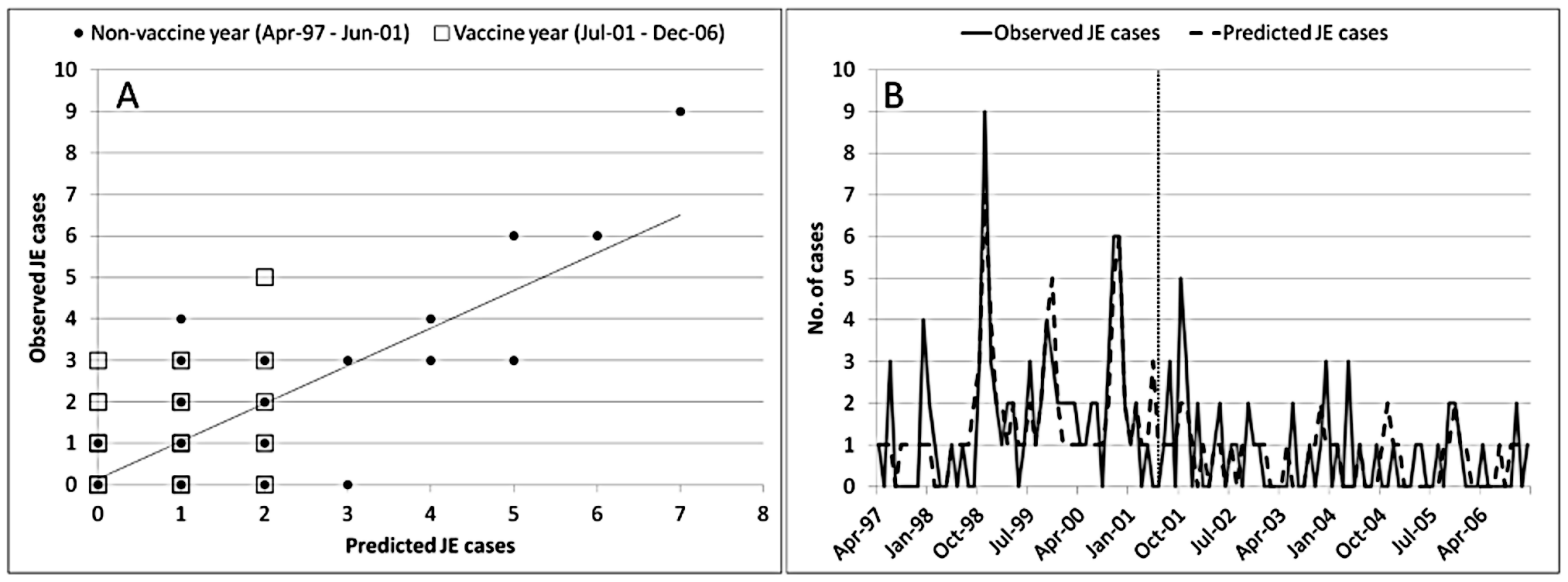
Observed versus predicted JE cases for observed climate and vaccine model (vaccine, seasonality, temperature, rainfall and SOI): time series (A); the vertical grey dotted line represents the introduction of the JE vaccine into the National Immunization program in Sarawak, Malaysia in July 2001 and scatter plot (B) of predicted versus observed values of JE cases; diagonal line represent the trend through all data points during the non-vaccine and vaccine years. Model fitted using Poisson regression.

The observed climate and vaccine model performed relatively well with a pseudo-R^2^ value of 0.370 suggesting an improvement 37.0% in the fitted model from the null (intercept only) model (Figure 2). Autocorrelation tests undertaken on the residuals of the fitted model to determine the goodness-of-fit showed no significant autocorrelation at different lags (see Supplemental Material, Figure S3).

### Prediction of JE cases without and with JE vaccination

**Figure 3:**
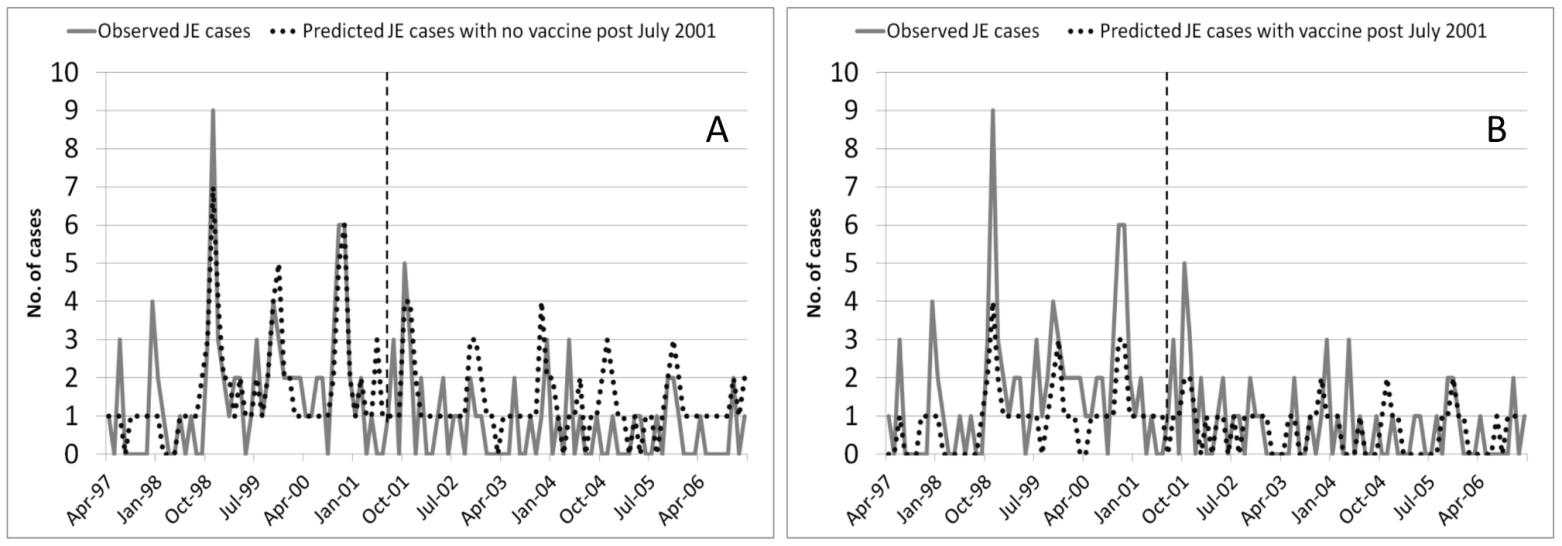
Observed versus predicted JE cases (A) without and (B) with vaccine coverage over the 10-year surveillance period: the vertical grey dotted line represents the introduction of the JE vaccine into the National Immunization program in Sarawak, Malaysia in July 2001. A–Predicted JE cases modelled without vaccine coverage over the 10-year period, minimum temperature at 6-months lag, rainfall at 1-month lag, SOI at 6-months lag and modelled seasonality. B– Predicted JE cases modelled with vaccine coverage throughout the 10-year surveillance period, minimum temperature at 6-months lag, rainfall at 1-month lag, SOI at 6-months lag and modelled seasonality).

Table 3 shows the sum of the observed and predicted JE cases modelled with and without vaccination over the 10-year surveillance period. The observed and predicted JE case sums were similar with the model slightly over-predicting the pre-vaccine period and under predicting the post-vaccine period and the total. When the model assumed no vaccine coverage over the 10-year surveillance period, 175 JE cases were predicted; when the model assumed vaccine coverage throughout the 10-year surveillance period, only 90 cases were predicted (Table 3). Figure 3 shows the time series of observed and predicted JE cases modelled with and without vaccination over the 10-year surveillance period.

**Table 3:** Sum of observed and predicted Japanese encephalitis cases modeled with and without vaccination over the 10-year surveillance period.

		Modeled		
Vaccine coverage	Observed JE cases	Predicted JE cases^1^	Predicted JE cases with no vaccine^2^	Predicted JE cases with vaccine^3^
**Pre-vaccine (Apr-97–Jun-01)**	84	87	87	48
**Post-vaccine (Jul-01–Dec-06)**	49	43	88	42
**Total**	133	130	175	90

1 Predicted JE cases are predictions where the vaccine regime was as observed.

2 Predicted JE cases with no vaccine assume no vaccine over the entire 10-years period.

3 Predicted JE cases with vaccine assume vaccine usage over the entire 10-years period.

### Vaccine effectiveness sensitivity analysis

**Figure 4:**
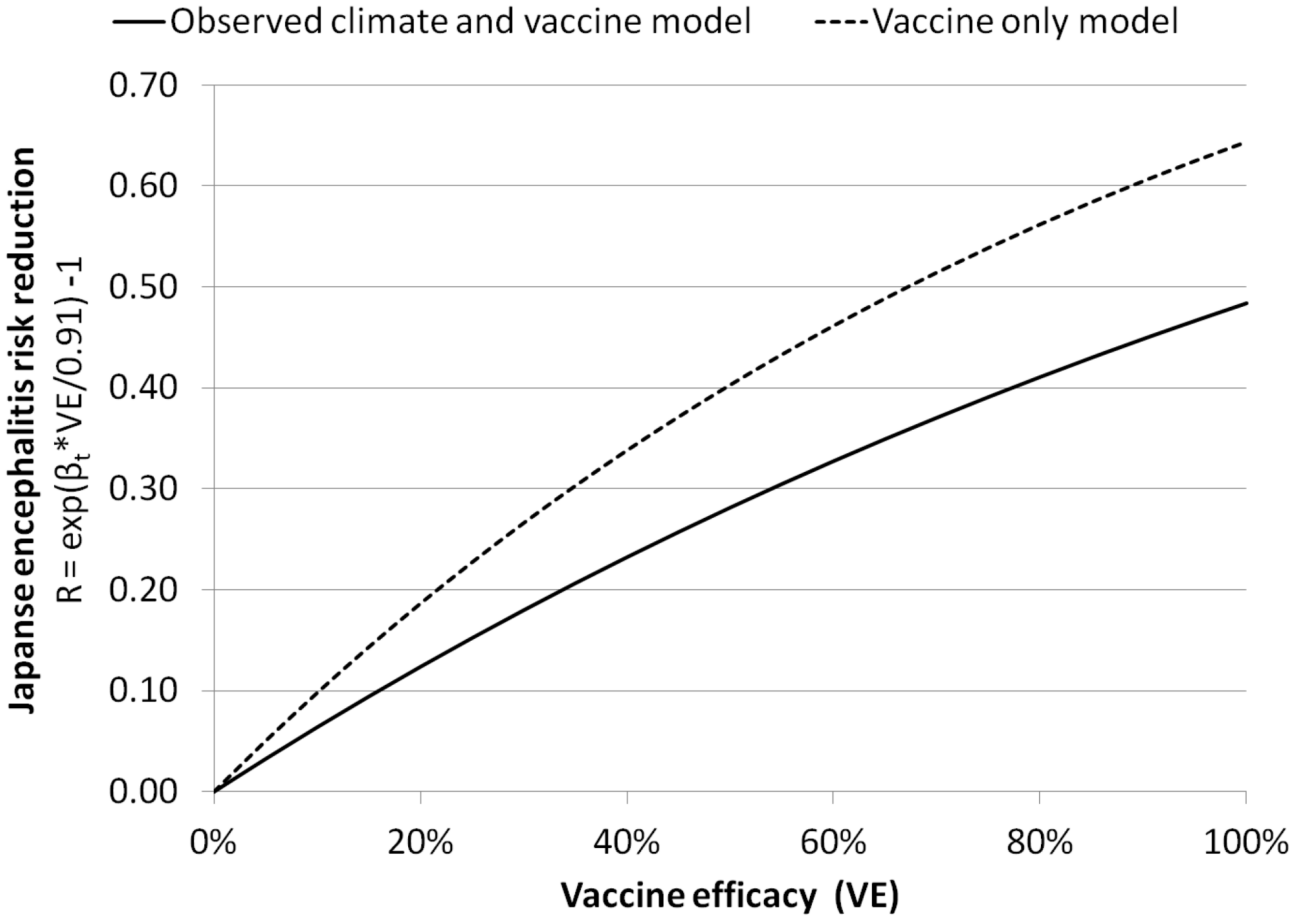
Japanese encephalitis risk reduction at different vaccine efficacies (VE) for the vaccine only model (vaccine, seasonality and no weather variables), and the observed climate and vaccine model (vaccine, seasonality, temperature, rainfall and SOI).

Figure 4 shows the JE risk reduction at different vaccine efficacies (VE) for the vaccine only model and observed climate and vaccine model. In the vaccine only model, the sensitivity analysis shows that ranges in the risk reduction of JE cases due to the vaccine programme is approximately between 45 and 65% for vaccine efficacies ranging between 60 and 100% under the current vaccine strategy in Sarawak. However, the observed climate and vaccine model shows a lower risk reduction of JE cases due to the vaccine programme. The observed climate and vaccine model with SOI shows a risk reduction of approximately 30 to 45% for vaccine efficacies ranging between 60 and 100%.

## Discussion

This study is the first to use laboratory-confirmed human JE cases to investigate the impact of vaccine, climate and ENSO on JEV transmission in Sarawak, Malaysia, a JEV endemic area. We provided an improved estimate of the reduction in JE cases in Sarawak, Malaysia after the introduction of the JE vaccine into the national child vaccination program, by taking account of climate inter-annual variability. We also investigated the association between meteorological variables and JE cases in the presence of vaccine coverage as well as assessing statistical interaction and confounding among the variables.

Wong *et al* (2008) and Ooi *et al* (2008) fully describe the demographic characteristics of the patients pre- and post-vaccination [18], [19]. Briefly, one hundred and twenty-two (92%) patients were children aged 12 years and younger, 11 (8%) patients were older children and adults. After vaccination, the mean age (range) of all affected patients shifted from 7.0 to 9.2 years. This shift in age distribution has also occurred in other countries where JE vaccine was introduced, namely in Japan, Korea, China and Taiwan [33]. When we consider the study of Wong *et al* 2008 who looked at JE cases before and after the vaccination program, they report a 56% drop in JE cases in children ≤12 years of age over the 6-year period after the implementation of the vaccination programme (Non-vaccine years: 9.8 cases/10^5^/year vs. Vaccine years: 4.3 cases/10^5^/year) in July 2001; however, they also suggested that a ‘catch-up’ program was required since older children and teenagers were not included in the current vaccination programme [19]. The model developed in this study may be useful for temporally targeting this population.

Because the magnitude of vector-borne disease outbreaks are often regulated by climate, due to sensitivities of the vector to temperature, humidity and rainfall, the impact of vaccination programmes on disease risk reduction must be evaluated carefully. There was a difference in the magnitude of risk reduction in JE cases due the vaccination programme in vaccine only model (RR: 0.391) and the observed climate and vaccine models (RR: 0.548). This study may be useful to other JE-endemic countries that have not implemented JE vaccination programmes and have increasing trend in JE incidence such as Indonesia, Cambodia, and Bangladesh [34] or are in the early stages of their vaccination programme such as India [35]. Given climate change, these countries should consider what will be the true impact of their vaccination programme. Furthermore, because vaccine efficacy can be influenced by various operational issues [36], we assessed a range of vaccine efficacy estimates to determine the array of JE case reductions that could be achieved. We found that even a 60% vaccine efficacy would result in greater than 30% risk reduction in JE cases in Sarawak after controlling for climate. This analysis shows the robustness of JE vaccination even in the face of climate. Therefore, taking full account of climate, the effect of the current Malaysian vaccine strategy is to reduce cases by approximately 50% (i.e. 88 saved out of 175).

Vaccine coverage, minimum temperature, rainfall, and SOI provided the best fit to JE case data. Surprisingly, we found the 6-month lag time between minimum temperature and JE cases to be highly significant, and this is consistent with some other studies that report 6-month lags. However, the full biological basis is unclear as it exceeds the lifespan of the vector. On the other hand, this statistical result does not imply causality. It may be confounded with other temperature variables at different lags which have direct biological effects. Still, JEV is transmitted year-round in Malaysia; such lag times may indeed describe the transmission pattern in the region.

SOI at 6-month lag had a strong positive association with JE cases and in our model. It is important to note that an increasing SOI would suggest a tendency towards a La Niña phase (prolonged long-term cooling in Pacific Ocean surface temperatures) while a decreasing SOI would suggest a tendency towards El Niño phase (prolonged long-term warming in Pacific Ocean sea surface temperatures). In 1997–1998, the largest El Niño event was observed. This was followed in 1998–2000, by a strong La Niña phase. El Niño phases are generally associated with warmer and drier than average climatic conditions, while La Niña phases are related to cooler and wetter climate conditions in Malaysia [37]. During the same period, there was a slightly higher level of reporting of JE in Sarawak, Malaysia during the high transmission peak of August to December.

Given our results on the impact of temperature, rainfall and SOI, on the occurrence of JE cases, further study would be useful to determine how they shape JEV transmission in Malaysia.

The four major limitation of this study were 1) highly focused and small scale nature of the original pilot hospital-based surveillance study, which only used a single hospital and a passive surveillance method; 2) the reporting bias from the relatively higher peak of JE cases in Sarawak in 1998, which also coincided with the Nipah virus outbreak in Malaysia and led to greater effort to accurately identify encephalitic cases relative to the other years; 3) a very limited time series (i.e. 10 years) during which there was only one relatively large JE outbreak peak, only 3 ENSO events (2 El Niño and 1 La Niña) and only 4 years when there was no vaccine; and 4) because West Nile (Kunjin) virus has been isolated from mosquitoes in Sarawak [38] patients may have been misdiagnosed due to cross-reactivity between West Nile virus (WN) virus (WNV) and JEV. Because we focused on a single hospital in Sarawak, which was the main referral hospital in the area, and collected accurate data, we are confident we have a less biased sample that is providing a lucid picture of JEV infection trends. A study demonstrated the high peak in 1998 during the Nipah outbreak was for the whole Sarawak state, while the peak for Sibu during the same period was not substantially different from the other years [19], suggesting that the data collected for Sibu are less prone to clinician awareness bias since even the heightened reporting for encephalitis cases in 1998/1999 did not change the reporting pattern in Sibu. With such a short time period and the reduction of disease cases with vaccination, it is difficult to say with certainty whether the patterns seen in the study are regular predictable phenomena or random events; therefore, longer studies with pig or mosquito JE incidence may be useful in future studies. Whilst the serological tests employed in the study were able to distinguish infections caused by dengue virus from JEV, it cannot do so for infections caused by WNV. Because of this limitation, we have conducted a limited serological study for WNV infection in patients who presented with acute encephalitis syndrome and found no evidence of West Nile infection in the patient group (unpublished data).

### Conclusion

Minimum temperature, rainfall and SOI were the key meteorological determinants associated with JE cases in Sarawak. These variables may be useful for developing an early-warning system based on seasonal climatic forecasting to temporally target vaccination programs to reduce the associated cost of vaccine distribution as well as scaling-up the current vaccine strategy to include un-prioritized or underserved populations. However, the mechanisms by which these variables impact JEV transmission is not entirely clear and require further investigation for development of a potential forecast system. For diseases like JE, vaccination for mitigation of disease cases should always be a top priority. However, to increase estimation accuracy of the vaccine effect, climate should be considered in epidemiological vaccine evaluations to determine the un-confounded impact of vaccination programs.

## Supporting Information

Figure S1 Map of study area in Malaysia.
(TIF)

Figure S2 Temporal variation in serologically confirmed JE cases from Sarawak from April 1997 to December 2006 and (A) mean temperature, (B) maximum temperature, (C) minimum temperature, (D) rainfall), (E) southern oscillation index climate and associated anomalies. The black vertical line represents the introduction of the JE vaccine into the National Immunization program in Sarawak, Malaysia in July 2001.
(TIF)

Figure S3 Goodness of fit graphs using residuals from observed climate and vaccine model (vaccine, seasonality, temperature, rainfall and SOI) model: Autocorrelation function-ACF (A) and sequence chart of raw residuals of the final model (B). Broken lines in the autocorrelation function (B) represent the 95% confidence limits (i.e. ± α-value 1.96 * standard error).
(TIF)

Table S1 Pearson's correlation between Sarawak Japanese encephalitis cases and climate variables at different lags.
(DOCX)

Table S2 Risk ratio of the upper-lower quartile difference for minimum temperature, rainfall and ENSO indices.
(DOCX)

## References

[1] Solomon T (2006) Control of Japanese encephalitis–within our grasp? N Engl J Med 355: 869–871. 16943399 10.1056/NEJMp058263

[2] Campbell GL, Hills SL, Fischer M, Jacobson JA, Hoke CH, et al (2011) Estimated global incidence of Japanese encephalitis: a systematic review. Bull World Health Organ 89: 766–774, 774A–774E. 22084515 10.2471/BLT.10.085233 PMC3209971

[3] Diagana M, Preux PM, Dumas M (2007) Japanese encephalitis revisited. J Neurol Sci 262: 165–170. 17643451 10.1016/j.jns.2007.06.041

[4] Umenai T, Krzysko R, Bektimirov TA, Assaad FA (1985) Japanese encephalitis: current worldwide status. Bull World Health Organ 63: 625–631. 3002650 PMC2536386

[5] Endy TP, Nisalak A (2002) Japanese encephalitis virus: ecology and epidemiology. Curr Top Microbiol Immunol 267: 11–48. 12082986 10.1007/978-3-642-59403-8_2

[6] Bi P, Tong S, Donald K, Parton KA, Ni J (2003) Climate variability and transmission of Japanese encephalitis in eastern China. Vector Borne Zoonotic Dis 3: 111–115. 14511580 10.1089/153036603768395807

[7] Bi P, Zhang Y, Parton KA (2007) Weather variables and Japanese encephalitis in the metropolitan area of Jinan city, China. J Infect 55: 551–556. 17714787 10.1016/j.jinf.2007.07.004

[8] Hsu SM, Yen AM, Chen TH (2008) The impact of climate on Japanese encephalitis. Epidemiol Infect 136: 980–987. 17767793 10.1017/S0950268807009454 PMC2870885

[9] Lin H, Yang L, Liu Q, Wang T, Hossain SR, et al (2011) Time series analysis of Japanese encephalitis and weather in Linyi City, China. Int J Public Health 57 2: 289–96. 21308477 10.1007/s00038-011-0236-x

[10] Philander SGH (1990) El Niño, La Niña and the Southern Oscillation. San Diego, CA: Academic Press. 10.1126/science.248.4957.904 17811864

[11] Bangs MJ, Larasati RP, Corwin AL, Wuryadi S (2006) Climatic factors associated with epidemic dengue in Palembang, Indonesia: implications of short-term meteorological events on virus transmission. Southeast Asian J Trop Med Public Health 37: 1103–1116. 17333762

[12] Corwin AL, Larasati RP, Bangs MJ, Wuryadi S, Arjoso S, et al (2001) Epidemic dengue transmission in southern Sumatra, Indonesia. Trans R Soc Trop Med Hyg 95: 257–265. 11490992 10.1016/s0035-9203(01)90229-9

[13] Fuller DO, Troyo A, Beier JC (2009) El Nino Southern Oscillation and vegetation dynamics as predictors of dengue fever cases in Costa Rica. Environ Res Lett 4: 140111–140118. 19763186 10.1088/1748-9326/4/1/014011 PMC2745182

[14] Tipayamongkholgul M, Fang CT, Klinchan S, Liu CM, King CC (2009) Effects of the El Nino-southern oscillation on dengue epidemics in Thailand, 1996–2005. BMC Public Health 9: 422. 19930557 10.1186/1471-2458-9-422 PMC2785791

[15] Earnest A, Tan SB, Wilder-Smith A (2012) Meteorological factors and El Nino Southern Oscillation are independently associated with dengue infections. Epidemiology and infection 140: 1244–1251. 21906411 10.1017/S095026881100183X

[16] Anyamba A, Chretien JP, Small J, Tucker CJ, Linthicum KJ (2006) Developing global climate anomalies suggest potential disease risks for 2006–2007. Int J Health Geogr 5: 60. 17194307 10.1186/1476-072X-5-60 PMC1779293

[17] Malaysian Meteorological Department (2012) Monsoon. [accessed 21 January 2013].

[18] Ooi MH, Lewthwaite P, Lai BF, Mohan A, Clear D, et al (2008) The epidemiology, clinical features, and long-term prognosis of Japanese encephalitis in central sarawak, malaysia, 1997–2005. Clin Infect Dis 47: 458–468. 18616397 10.1086/590008

[19] Wong SC, Ooi MH, Abdullah AR, Wong SY, Krishnan S, et al (2008) A decade of Japanese encephalitis surveillance in Sarawak, Malaysia: 1997–2006. Trop Med Int Health 13: 52–55. 18291002 10.1111/j.1365-3156.2007.01967.x

[20] Sarawak State Health Department (2002) The recommended immunization schedule for Sarawak. (Available at: http://wwwsarawakhealthgovmy/immunization1html) Accessed [21 January 2012].

[21] Sarawak State Health Department (2007) Epidemiological News. (Available at: http://wwwsarawakhealthgovmy/Images/Epid%20News%20JAN%202007%20CDCpdf) Accessed [21 January 2013].

[22] Cardosa MJ, Wang SM, Sum MS, Tio PH (2002) Antibodies against prM protein distinguish between previous infection with dengue and Japanese encephalitis viruses. BMC Microbiol 2: 9. 12019028 10.1186/1471-2180-2-9 PMC113253

[23] Solomon T, Thao LT, Dung NM, Kneen R, Hung NT, et al (1998) Rapid diagnosis of Japanese encephalitis by using an immunoglobulin M dot enzyme immunoassay. J Clin Microbiol 36: 2030–2034. 9650956 10.1128/jcm.36.7.2030-2034.1998 PMC104972

[24] KNMI Climate Explorer (1999) KNMI Climate Explorer. (Available at: http://wwwcruueaacuk/) [accessed 21 January 2013].

[25] University of East Anglia (2013) Climatic Research Unit. (Available at: http://wwwcruueaacuk/) [accessed at: 21 January 2013].

[26] Mitchell TD, Jones PD (2005) An improved method of constructing a database of monthly climate observations and associated high-resolution grids. International Journal of Climatology 25: 693–712.

[27] Ropelewski CF, Jones PD (1987) An Extension of the Tahiti-Darwin Southern Oscillation Index. Monthly Weather Review 115: 2161–2165.

[28] NOAA Climate Prediction Center (2012) Monthly Atmospheric & SST Indices (Available at: http://wwwcpcncepnoaagov/data/indices/) [accessed 21 January 2013].

[29] Centers for Disease Control and Prevention (1993) Inactivated Japanese encephalitis virus vaccine. Recommendations of the Advisory Committee on Immunization Practices (ACIP). MMWR Recomm Rep 42: 1–15. 8381504

[30] Hoke CH, Nisalak A, Sangawhipa N, Jatanasen S, Laorakapongse T, et al (1988) Protection against Japanese encephalitis by inactivated vaccines. N Engl J Med 319: 608–614. 2842677 10.1056/NEJM198809083191004

[31] Chiang J, Sobel A (2002) Tropical tropospheric temperature variations caused by ENSO and their influence on the remote tropical climate. Journal of Climatology 15 2616–2631.

[32] Linthicum KJ, Anyamba A, Chretien JP, Small J, Tucker CJ, et al.. (2010) The role of global climate patterns in the spatial and temporal distribution of vector-borne disease. In: Atkinson PW, editor. Vector Biology, Ecology, and Control: Springer.

[33] Tsai TF, Chang GJ, Yu YX (1999) Japanese encephalitis vaccines. In: Plotkin SA, Orenstein WA, editors. Vaccines. WB Saunders Company. pp. 672–710.

[34] Erlanger TE, Weiss S, Keiser J, Utzinger J, Wiedenmayer K (2009) Past, present, and future of Japanese encephalitis. Emerg Infect Dis 15: 1–7. 19116041 10.3201/eid1501.080311 PMC2660690

[35] Vashishtha VM (2012) Status of immunization and need for intensification of routine immunization in India. Indian pediatrics 49: 357–361. 22700663 10.1007/s13312-012-0081-x

[36] Comstock GW (1994) Evaluating vaccination effectiveness and vaccine efficacy by means of case-control studies. Epidemiol Rev 16: 77–89. 7925730 10.1093/oxfordjournals.epirev.a036147

[37] Glantz MH (2001) Currents of Change: El Niño's Impact on Climate and Society. Cambridge: Cambridge University Press.

[38] Ching CY, Casals J, Bowen ET, Simpson DI, Platt GS, et al (1970) Arbovirus infections in Sarawak: the isolation of Kunjin virus from mosquitoes of the Culex pseudovishnui group. Ann Trop Med Parasitol 64: 263–268. 5500097 10.1080/00034983.1970.11686690

