# Supplementary figures and images for: The Effect of Vaccination Coverage and Climate on Japanese Encephalitis in Sarawak, Malaysia
Source: PLoS Negl Trop Dis. 2013 Aug 8;7(8):e2334. doi: 10.1371/journal.pntd.0002334 (PMC3738455; doi:10.1371/journal.pntd.0002334)

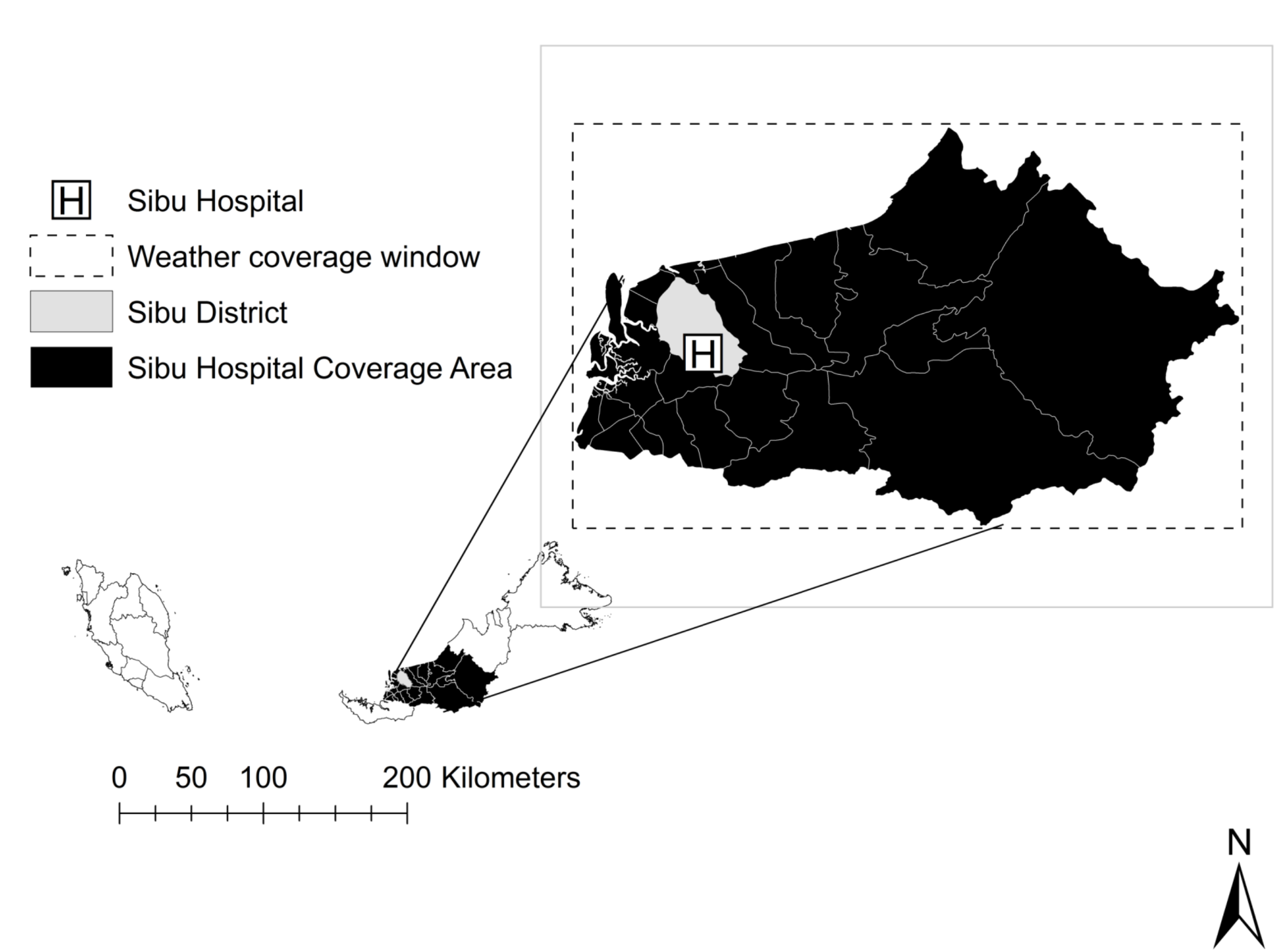

Supplement: Figure S1 — Map of study area in Malaysia. (TIF) [file pntd.0002334.s001.tif]

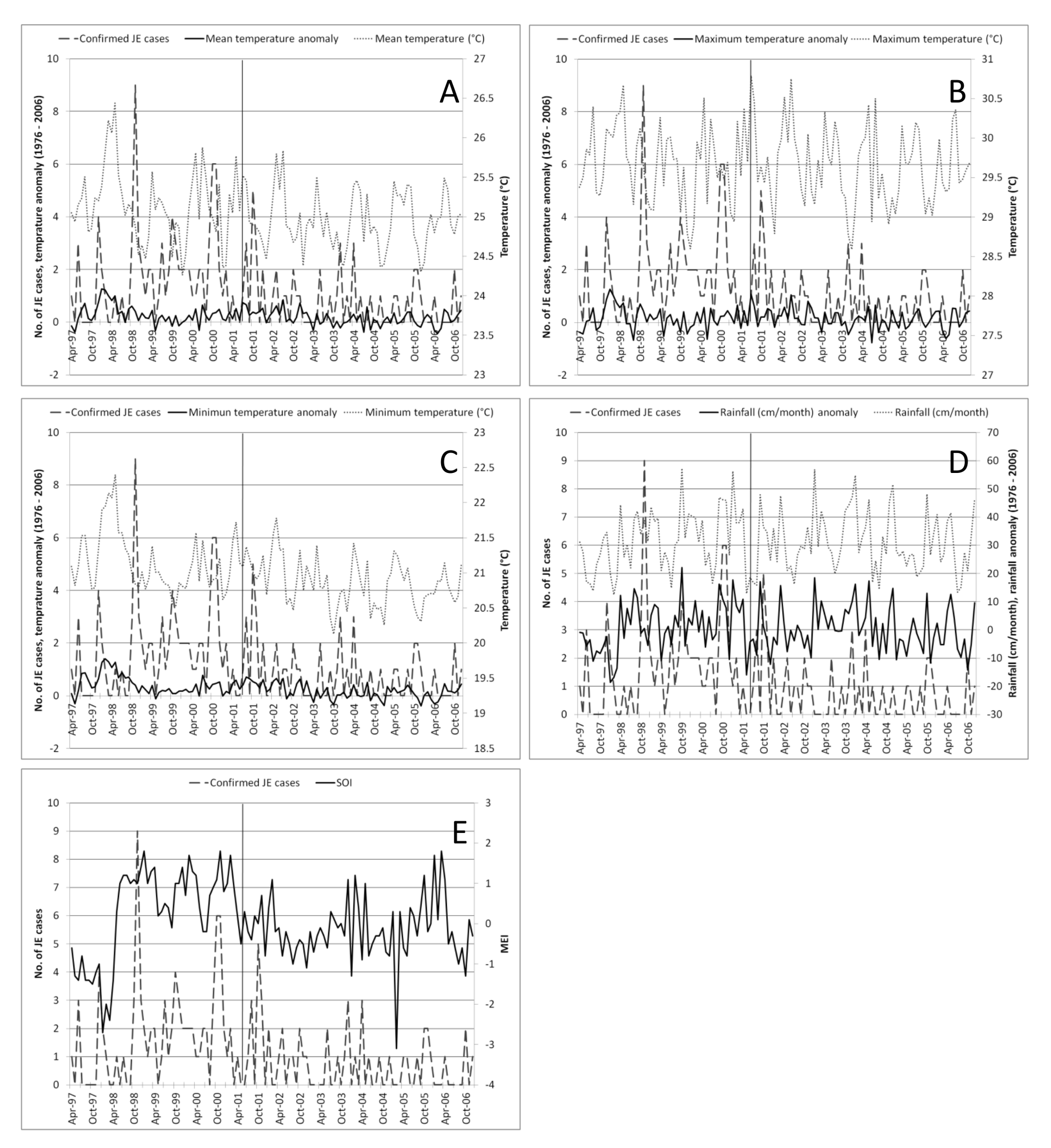

Supplement: Figure S2 — Temporal variation in serologically confirmed JE cases from Sarawak from April 1997 to December 2006 and (A) mean temperature, (B) maximum temperature, (C) minimum temperature, (D) rainfall), (E) southern oscillation index climate and associated anomalies. The black vertical line represents the introduction of the JE vaccine into the National Immunization program in Sarawak, Malaysia in July 2001. (TIF) [file pntd.0002334.s002.tif]

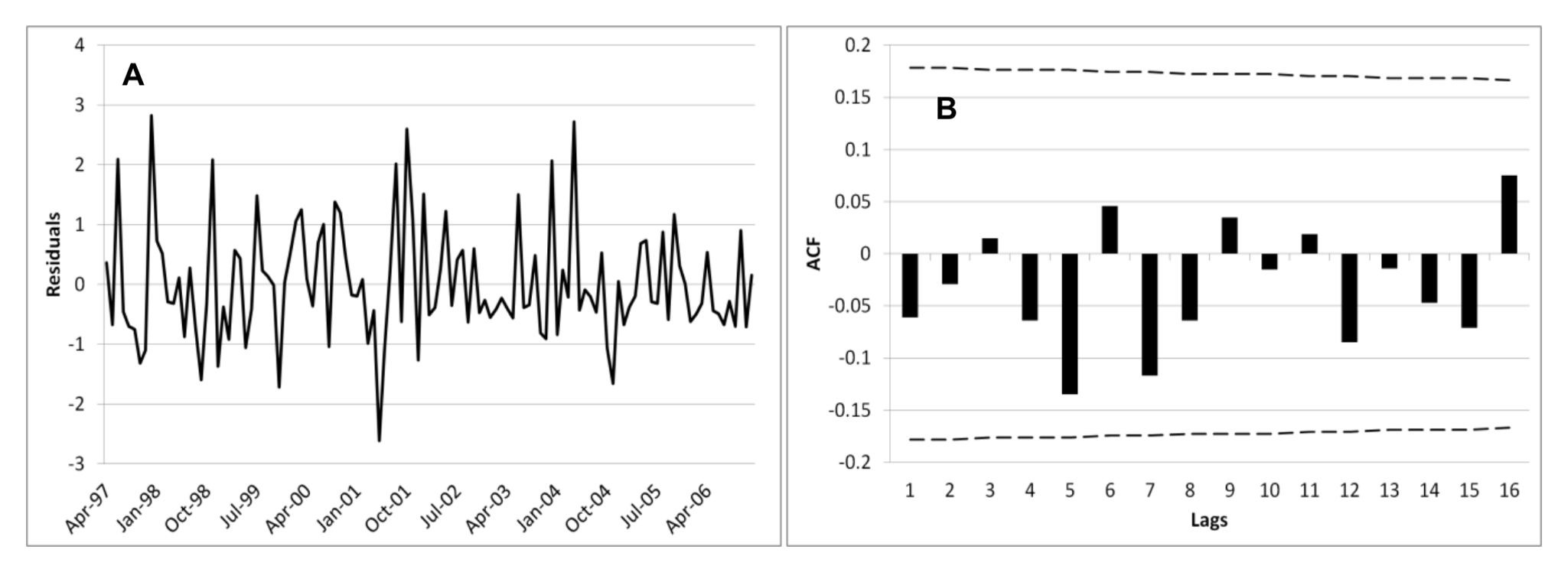

Supplement: Figure S3 — Goodness of fit graphs using residuals from observed climate and vaccine model (vaccine, seasonality, temperature, rainfall and SOI) model: Autocorrelation function-ACF (A) and sequence chart of raw residuals of the final model (B). Broken lines in the autocorrelation function (B) represent the 95% confidence limits (i.e. ± α-value 1.96 * standard error). (TIF) [file pntd.0002334.s003.tif]
