# Supplementary material for: The Effect of Vaccination Coverage and Climate on Japanese Encephalitis in Sarawak, Malaysia
Source: PLoS Negl Trop Dis. 2013 Aug 8;7(8):e2334. doi: 10.1371/journal.pntd.0002334 (PMC3738455; doi:10.1371/journal.pntd.0002334)
Supplement: Table S1 — Pearson's correlation between Sarawak Japanese encephalitis cases and climate variables at different lags. (DOCX) [file pntd.0002334.s004.docx]

Supplemental Material - **The effect of vaccination coverage and climate on Japanese encephalitis in Sarawak, Malaysia** - Daniel E. Impoinvil, Mong How Ooi, Peter J. Diggle, Cyril Caminade, Mary Jane Cardosa, Andrew P. Morse, Matthew Baylis and Tom Solomon

| Supplemental Material, Table S1. **Pearson’s correlation between Sarawak Japanese encephalitis cases and climate variables at different lags** | | | | |
| --- | --- | --- | --- | --- |
|  | **Temperature** | | |  |
| **Lags** | **Mean** | **Maximum** | **Minimum** | **Rainfall** |
| 0 | -0.099 | -0.040 | -0.045 | 0.206* |
| 1 | -0.037 | 0.0290 | -0.103 | 0.300** |
| 2 | 0.060 | 0.000 | -0.029 | 0.072 |
| 3 | 0.149 | 0.109 | 0.102 | -0.116 |
| 4 | 0.120 | 0.066 | 0.171 | -0.135 |
| 5 | 0.221* | 0.095* | 0.245** | -0.137 |
| 6 | 0.356** | 0.240** | 0.391** | -0.047 |
| 7 | 0.279** | 0.180** | 0.269* | 0.104 |
| 8 | 0.123 | -0.032 | 0.216* | 0.030 |
| 9 | -0.006 | -0.083 | 0.197 | 0.024 |
| 10 | -0.070 | -0.144 | 0.095 | 0.073 |
| 11 | -0.020 | -0.044 | 0.039 | 0.147 |
| 12 | -0.048 | -0.079 | -0.056 | 0.209* |
| *p<0.05, **p<0.01 | | | | |
