# Supplementary material for: The Effect of Vaccination Coverage and Climate on Japanese Encephalitis in Sarawak, Malaysia
Source: PLoS Negl Trop Dis. 2013 Aug 8;7(8):e2334. doi: 10.1371/journal.pntd.0002334 (PMC3738455; doi:10.1371/journal.pntd.0002334)
Supplement: Table S2 — Risk ratio of the upper-lower quartile difference for minimum temperature, rainfall and ENSO indices. (DOCX) [file pntd.0002334.s005.docx]

Supplemental Material - **The effect of vaccination coverage and climate on Japanese encephalitis in Sarawak, Malaysia** - Daniel E. Impoinvil, Mong How Ooi, Peter J. Diggle, Cyril Caminade, Mary Jane Cardosa, Andrew P. Morse, Matthew Baylis and Tom Solomon

| Supplemental Material, Table S2. Risk ratio of the upper-lower quartile difference for minimum temperature, rainfall and ENSO indices | | | |
| --- | --- | --- | --- |
| **Quartile** | **Minimum**  **temperature at 6-months lag** | **Rainfall**  **(cm/month) at 1-month lag** | **SOI at 6-months lag** |
| Lower: 25 | 20.7081 | 22.9968 | -0.5 |
| Median: 50 | 20.897 | 29.2113 | 0 |
| Upper: 75 | 21.1998 | 38.7943 | 1 |
|  |  |  |  |
| Upper-Lower  Quartile Difference | 0.4917 | 15.7975 | 1.5 |
|  |  |  |  |
| SOI –model | **RR** |  |  |
| Min. temp. (°C) | 1.41 |  |  |
| Rainfall | 1.53 |  |  |
| SOI | 1.66 |  |  |
| Risk ratio = RR = exp(β_t_*q_3_-q_1_) where β_t_ is the slope of explanatory variable from the model (see table 3) and q_1_ and q_3_ is the upper-lower quartile difference | | | |
